# Cocaine-induced renal infarction: report of a case and review of the literature

**DOI:** 10.1186/1471-2369-6-10

**Published:** 2005-09-22

**Authors:** Shahrooz Bemanian, Mazda Motallebi, Saeid M Nosrati

**Affiliations:** 1 Chief Resident, Department of Medicine, University of Southern California, Keck School of Medicine, LAC-USC Medical Center, Los Angeles, USA; 2 Clinical Fellow, Department of Medicine, Division of Cardiovascular Medicine, University of Southern California, Keck School of Medicine, LAC-USC Medical Center, Los Angeles, USA; 3 Associate Professor of Clinical Medicine, Department of Medicine, Division of Nephrology, University of Southern California, Keck School of Medicine, LAC-USC Medical Center, Los Angeles, USA

## Abstract

**Background:**

Cocaine abuse has been known to have detrimental effects on the cardiovascular system. Its toxicity has been associated with myocardial ischemia, cerebrovascular accidents and mesenteric ischemia. The pathophysiology of cocaine-related renal injury is multifactorial and involves renal hemodynamic changes, alterations in glomerular matrix synthesis, degradation and oxidative stress, and possibly induction of renal atherogenesis. Renal infarction as a result of cocaine exposure, however, is rarely reported in the literature.

**Case presentation:**

A 48 year-old male presented with a four-day history of severe right flank pain following cocaine use. On presentation, he was tachycardic, febrile and had severe right costovertebral angle tenderness. He had significant proteinuria, leukocytosis and elevated serum creatinine and lactate dehydrogenase. Radiographic imaging studies as well as other screening tests for thromboembolic events, hypercoagulability states, collagen vascular diseases and lipid disorders were suggestive of Cocaine-Induced Renal Infarction (CIRI) by exclusion.

**Conclusion:**

In a patient with a history of cocaine abuse presenting with fevers and flank pain suggestive of urinary tract infection or nephrolithiasis, cocaine-induced renal infarction must be considered in the differential diagnosis. In this article, we discuss the prior reported cases of CIRI and thoroughly review the literature available on this disorder. This is important for several reasons. First, it will allow us to discuss and elaborate on the mechanism of renal injury caused by cocaine. In addition, this review will demonstrate the importance of considering the diagnosis of CIRI in a patient with documented cocaine use and an atypical presentation of acute renal injury. Finally, we will emphasize the need for a consensus on optimal treatment of this disease, for which therapy is not yet standardized.

## Background

Cocaine abuse has been known to have detrimental effects on the cardiovascular system. Its toxicity has been associated with myocardial ischemia, cerebrovascular accidents and mesenteric ischemia. The pathophysiology of cocaine-related renal injury is multifactorial and involves renal hemodynamic changes, alterations in glomerular matrix synthesis, degradation and oxidative stress, and possibly induction of renal atherogenesis. Renal infarction as a result of cocaine exposure, however, is rarely reported in the literature.

## Case presentation

**Figure 1:**
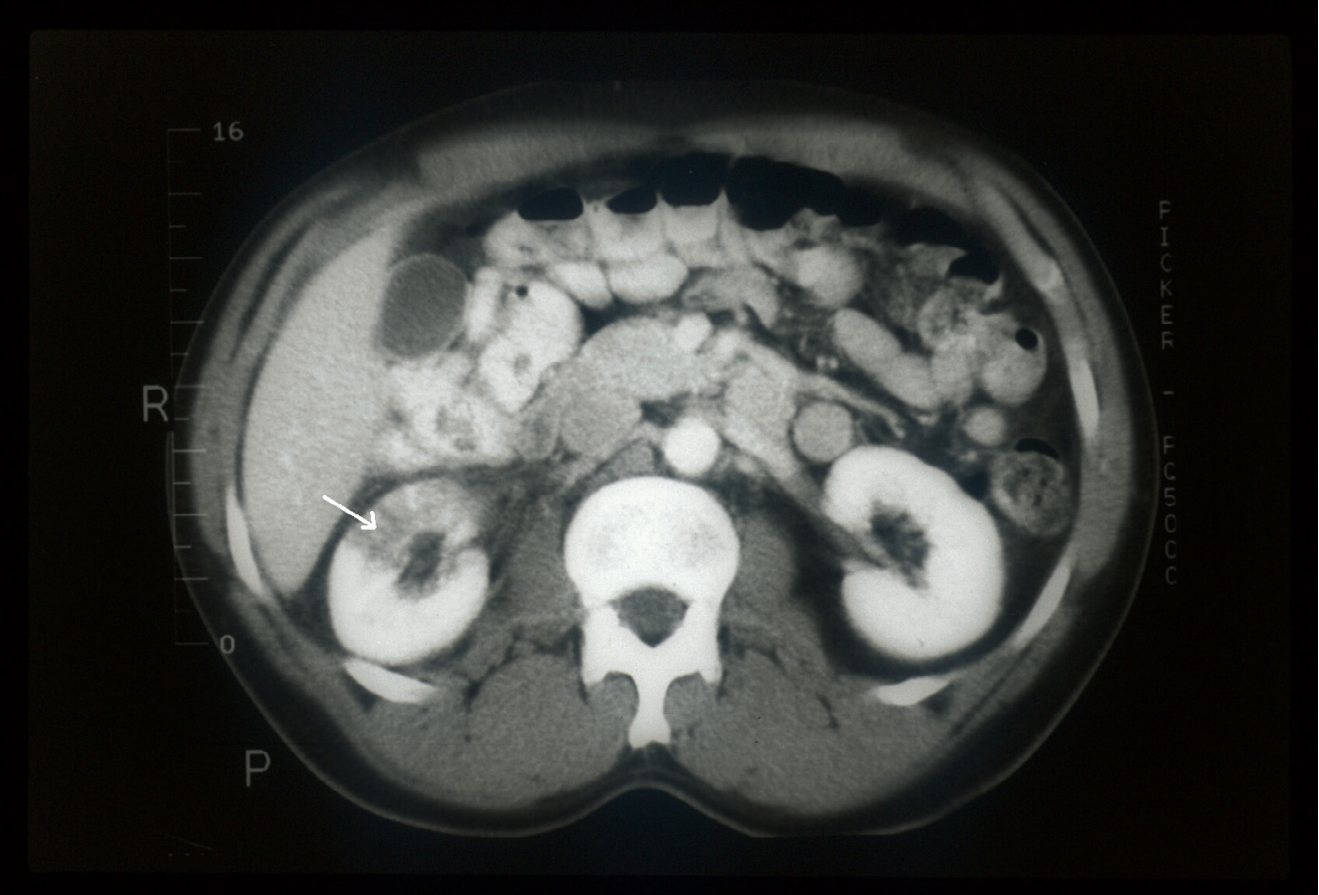
Computerized Tomography showing areas of focal decreased enhancement of the anterior lower poles (white arrow) of the right kidney suggesting renal infarction versus pyelonephritis.

**Figure 2:**
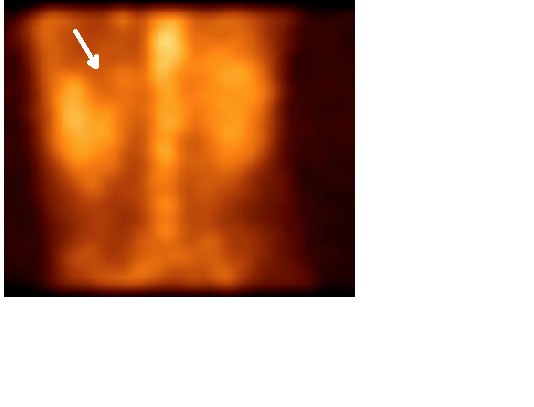
Volume-rendered Single Photon Emission-Computed Tomography image of a gallium scan demonstrating absence of tracer localization in the upper pole of the right kidney (white arrow) compatible with infarction.

A 48 year-old African American male presented to our hospital with a four-day history of severe right flank pain starting several hours after smoking cocaine. His pain was associated with subjective fevers, nausea and vomiting. There was no gross hematuria or dysuria. His blood pressure was 140/78 mmHg, heart rate 130 beats per minute, temperature 39.5 °C, respiratory rate 18 breaths per minute and his oxygen saturation was 98% on room air. Pertinent exam findings included severe right costovertebral angle tenderness, diffuse right-sided abdominal pain and a positive psoas sign on the right. Laboratory data showed a white blood cell count of 14 × 10^9^/L (76% neutrophils), serum creatinine 1.4 mg/dL (124 μmol/L), lactate dehydrogenase (LDH) 704 U/L and serum creatinine kinase (CPK) 259 U/L. A urinalysis showed 3+ proteinuria, 1 to 3 red blood cells per high power field and no white blood cells or casts. Urine toxicology confirmed the presence of cocaine. A Computerized Tomography (CT) scan with contrast revealed a sharply-demarcated heterogenous area of focal decreased enhancement in the anterior right kidney consistent with renal infarct, but pyelonephritis could not be completely ruled out (figure 1). Volume-rendered Single Photon Emission-Computed Tomography (SPECT) image of a gallium scan showed no evidence of tracer localization in the right kidney to suggest an infection. It did reveal absence of tracer localization in the upper pole of the right kidney suggesting absence of perfusion to that area (figure 2). Blood and urine cultures were negative. A trans-thoracic and a subsequent trans-esophageal echocardiogram showed no evidence of intra-cardiac thrombus or valvular vegetations. Further screening tests for hypercoagulability (factor V Leiden, prothrombin gene, protein C and protein S, anti-thrombin III, anti-phospholipid antibody and homocysteine); collagen vascular disease (rheumatoid factor and antinuclear antibody); and lipid disorders were within normal limits. Our patient was then diagnosed with CIRI by exclusion.

## Discussion

Cocaine abuse and addiction continues to be a problem that plagues the entire world. Cocaine (benzoyl methylecgonine) is available in two forms: cocaine hydrochloride and the alkaloid cocaine (freebase/crack), which is made by alkanizing the salt, followed by extraction with non-polar solvents. Both forms are well absorbed through most mucous membranes [1]. The plasma half-life is approximately 45 to 90 minutes. The elimination of cocaine is predominantly controlled by its biotransformation since its renal clearance is only 27 mL/min [2]. The pathways for the biotransformation involve plasma and liver cholinesterases that produce benzoyl and ethyl methylecgonine which are water-soluble metabolites excreted in urine [3].

The pathophysiologic effects of cocaine-induced renal injury involve several mechanisms. First, cocaine affects vascular reactivity and renal hemodynamics. This is thought to be due to its ability to inhibit uptake of catecholamines in the synapse, inhibit re-uptake of norepinephrine in sympathetically innervated tissues and release norepinephrine and epinephrine from the adrenal medulla [4,5]. There's also evidence that cocaine can directly increase calcium influx in vascular smooth muscle [6,7]. This effect has been documented in post-sympathectomy arteries as well as in umbilical arteries, which lack sympathetic innervation [6,7]. Second, cocaine has been shown to affect matrix synthesis degradation and oxidative stress in kidney [8]. Third, cocaine has been shown to accelerate renal atherogenesis [9,10].

Although major toxic effects of cocaine such as myocardial ischemia, cerebrovascular accidents, mesenteric ischemia and placental infarcts have been well- documented in the literature, renal infarction as a complication of cocaine toxicity is rare. In this article, we will attempt to raise awareness of this under-reported entity by discussing its pathophysiology, clinical presentation and the current treatment modalities.

Common risk factors for renal infarction include valvular heart disease and atheroembolic events. Hypercoagulopathy, systemic vasculitis, blunt trauma, collagen vascular disorders, vascular intervention procedures and endocarditis are other less common risk factors associated with renal infarct.

Cocaine-induced renal damage is well-documented in the literature; however, renal infarction due to cocaine is not a well-known entity. Throughout our literature search, we identified seven other such reports (table 1) [11-17]. In one patient, a renal infarct was thought to be due to unilateral right renal artery thrombosis and embolization associated with intravenous cocaine injection [11]. Kramer et al. described a patient with a right renal thrombosis and infarction associated with protein C deficiency [12]. In another report, Antonovych et al. noted unusual renal involvement in two cocaine addicts [13]. One patient was a 26 year-old male who presented with malignant hypertension, who was found to have renal infarction. From this report, it is uncertain whether cocaine-induced malignant hypertension led to renal infarction or if cocaine-induced renal infarction presented as a case of malignant hypertension. The other patient was a 39 year-old male with recent cocaine use who had an emergent surgical nephrectomy for unclear reasons, and was found to have arterial and venous thrombosis with massive infarction. Two other cases describe right renal infarctions in patients with recent cocaine use in whom no other abnormalities were found [14,15]. Edmondson et al. described a 40-year-old male with renal artery dissection and thrombosis presumed to be due to cocaine which led to renal infarction [16]. Mochizuki et al. reported a 52-year-old female with recent intranasal use of cocaine who presented with acute aortic thrombosis associated with renal infarct [17].

It is not known whether there is a gender predilection for this disorder. Interestingly, all but one of the reported cases of CIRI has occurred in males. This occurrence may be due to an increased prevalence of cocaine use in males. It is also possible that males may be more genetically prone to this disorder. However, given the small number of reported cases, one cannot make any certain conclusions about genetic susceptibility.

In our review, we also noted that all but one of the reported cases of CIRI have involved the right kidney. It is generally accepted that the right renal artery is longer than the left due to its anatomical position in relation to the great vessels. Merklin et al. investigated the varied blood supply of the kidneys by dissecting 185 kidneys obtained from adult cadavers. The length of the right renal artery from the aortic origin to its division point was as long as 8.0 cm and that of the left varied from 0.5 to 6.0 cm [18]. The calibers of these arteries were similar in diameter, and their average diameter was 5.5 mm with variations from 4 to 7 mm [18]. One must keep in mind that neither p-values nor confidence intervals were described in this study.

Steady flow models for blood flow analysis in human circulation are very complex. The simplest model for blood flow through any vessel assumes a flow of a fluid with a constant viscosity through a non-tortuous and straight cylindrical tube of circular cross-section [19]. Such fluid flows are well-described by Poiseuille Equations in the honor of J. L. M. Poiseuille who performed many experiments relating pressure gradient, flow, tube geometry and length [19].

Poiseuille formulated the factors that affect flow of fluid in a tube as:

Q = (P_1_-P_2_) π r^4^/8ήL

(Q = flow rate; P_1_- P_2 _= pressure difference across a circuit; r = radius; ή = viscosity of fluid; L = length of vessel).

Given the inverse relationship of flow to resistance:

Resistance = flow ^-1^

We can then conclude that resistance in a vessel is directly proportional to its length, and indirectly proportional to the radius to the power of four:

Resistance α Length/radius^4^

Since the radius of the right and left renal arteries are similar in size, we postulate that the right kidney is more prone to ischemia due to the increased resistance that it encounters by the longer length of its artery. Whether this is true or not, one must take into account the limited number of reported cases along with the lack of advanced studies to investigate the exact anatomy of the renal arteries in these particular patients.

The pathophysiology of cocaine-related renal injury is multifactorial and involves renal hemodynamic changes, alterations in glomerular matrix synthesis, degradation and oxidative stress, and possible induction of renal atherogenesis [8-10,20]. The exact mechanism of how cocaine causes renal infarction, however, is unclear, and several possibilities have been proposed. Cocaine is known to enhance platelet aggregation and increase thromboxane synthesis [21]. It is also known to inhibit synaptosomal uptake of catecholamines, block the re-uptake of norepinephrine in sympathetically innervated tissues and release norepinephrine and epinephrine from the adrenal medulla [4,5,21]. Other vasoconstrictive factors such as endothelin have also been postulated to play a role in the vascular catastrophes caused by cocaine intoxication [22].

There is evidence that cocaine may enhance the renal cortical messenger RNA expression of tissue inhibitors of metalloproteinase-2 [8]. This would have the net effect of increasing matrix accumulation. Use of cocaine also increases the oxidative stress in the kidney. In experimental cultured kidney cells exposed to cocaine, there has been a lower level of intracellular glutathione, which is the most abundant cell thiol with antioxidant functions [23]. There is also supporting evidence that systemic atherosclerosis may be an inflammatory disorder. Both experimental and autopsy findings confirm that cocaine is an accelerator of atherogenesis [9,10].

Renal infarction due to cocaine abuse usually presents with severe persistent flank and/or abdominal pain associated with nausea or vomiting with or without elevated temperature. The onset of pain is usually 2–3 hours after cocaine use, but it can be delayed for up to 4 days. All routes of cocaine administration including intravenous, insufflations, and intranasal and free-basing crack cocaine have been associated with renal infarction. Leukocytosis, microscopic hematuria and elevated levels of serum LDH are common findings. However, these findings are neither sensitive nor specific for CIRI.

Various imaging techniques including CT scan, Magnetic Resonant Imaging (MRI), angiography, Ultrasound and Nuclear Scintigraphy Scans have been used to make the diagnosis. However, MRI, angiography and nuclear scintigraphy are expensive, system-specific and time-consuming, and they may not be readily available at all institutions. Ultrasound lacks sensitivity and specificity for renal infarction. Therefore, CT scan has been recommended for the diagnosis of this challenging disease. CT scan, however, becomes less reliable when the infarct is global and there is loss of viable cortical rim, or when the infarct simulates a tumefactive process.

There is no consensus on the treatment of renal infarction due to cocaine use. Prior treatment modalities in the literature range from no treatment to anticoagulation, thrombolytic use, aspirin therapy and surgical nephrectomy. Given that no evidence of underlying coagulopathy or thromboembolic events were identified in our patient, we opted to conservatively manage him with supportive therapy and pain management. He was successfully discharged on hospital day six with a serum creatinine of 1.2 mg/dL (106.1 umol/L).

## Conclusion

Cocaine intoxication is associated with multiple cases of vascular injury. Renal infarction as a result of cocaine use, however, is uncommon. In a patient with a history of cocaine abuse presenting with fevers and flank pain suggestive of urinary tract infection or nephrolithiasis, cocaine-induced renal infarction must be considered in the differential diagnosis. The vasoconstrictive and thrombotic effects of cocaine are most likely the dominant factors in cocaine-induced renal infarction. The possible atherogenic effect of cocaine and the relationship of this effect to renal infarct is likely to be a long term, rather than an acute factor. The diagnosis of CIRI is based on exclusion of other entities that cause renal infarct (e.g. hypercoagulable states due to factor deficiency, autoimmune diseases and thromboembolic events), in conjunction with documented cocaine use and appropriate radiographic studies. We highly recommend non-invasive evaluation of the renal arterial supply in patients with CIRI in order to broaden our knowledge of this disorder, and conceivably better understand its selectivity for one kidney. Thus far, there is no agreement on the management of patients with this disease. In fact, it is difficult to suggest a uniform therapeutic approach to this disorder because one cannot predict whether the infarct will be due to vasospasm, thrombosis or both. In addition, the extent of the renal infarct will likely influence clinical management and outcome. Although anticoagulation, antithrombotic, conservative managements as well as surgical approaches have been attempted with variable success, perhaps the most plausible treatment would be to prevent this misfortune by patient education.

## Competing interests

The author(s) declare that they have no competing interests.

## Authors' contributions

SB performed the main background search and drafted the manuscript. MM participated in the study design and helped to draft the manuscript. SMN participated in the study design and coordination and helped to draft the manuscript. All authors read and approved the final manuscript.

## Pre-publication history

The pre-publication history for this paper can be accessed here:



## Figures and Tables

**Table 1:** Demographic data of previously-reported cases of cocaine-induced renal infarction.

Characteristics	Wohlman [11]	Antonovych [13]	Kramer [12]	Goodman [14]	Saleem [15]	Edmondson [16]	Mochizuki [17]
Age (s)	32	39, 26	37	37	25	40	52
Sex	Male	Males	Male	Male	Male	Male	Female
Route	IV	NR	NR	IN	IN	NR	IN
Time of Onset	2 hours	NR	2–3 hours	2 hours	4 days	1 day	NR
Kidney Affected	Right	NR	Right	Right	Right	Right	Left
WBC (× 10^3^/μl)	14	NR	23 (8% bands)	16 (90% PMN)	12.3 (76% PMN)	NR	19.3
Serum LDH (U/L)	368 to 1860	NR	2103	NR	351	NR	2100
Serum Cr (mg/dL)	1.2	NR	1.7	0.9	NR	1.3	0.9
Urinanalysis	5–10 RBC	NR	trace protein 3+ ketones 2+ blood 3–4 WBC/hpf 1–2 RBC/hpf	30 protein 80 ketones 1–5 WBC/hpf 5–10 RBC/hpf	2+ blood 2 WBC/hpf 5–10 RBC/hpf	0 RBC/hpf	trace protein large blood 3 WBC/hpf 1 RBC/hpf
Treatment	heparin & coumadin	nephrectomy	urokinase, heparin & coumadin	none	aspirin	heparin & coumadin	heparin, antibiotics & coumadin
Co-existing Conditions	NR	NR	DM, Protein C deficiency	NR	NR	Renal artery dissection	Aortic thrombosis

WBC (White Blood Cell; cells × 10^3^/ul = cells × 10^9^/ul); RBC (Red Blood Cell); hpf (High Power Field); LDH (Lactate Dehydrogenase); PMN (Polymorphonuclear Cells); N/R (Not Reported); IV (Intravenous); IN (Intra-nasal); DM (Diabetes Mellitus); Serum Cr (Serum Creatinine; 1 mg/dL = 88.4 umol/L)
